# Is the association of birth weight with premenopausal breast cancer risk mediated through childhood growth?

**DOI:** 10.1038/sj.bjc.6601972

**Published:** 2004-07-13

**Authors:** I dos Santos Silva, B L De Stavola, R J Hardy, D J Kuh, V A McCormack, M E J Wadsworth

**Affiliations:** 1 Department of Epidemiology and Population Health, London School of Hygiene and Tropical Medicine, Keppel Street, London WC1E 7HT, UK; 2 MRC National Survey of Health and Development, Department of Epidemiology and Public Health, University College, London WC1E 6BT, UK

**Keywords:** breast cancer, birth weight, height, body mass index, growth, menarche

## Abstract

Several studies have found positive associations between birth weight and breast cancer risk at premenopausal ages. The mechanisms underlying this association are not known, but it is possible that it may be mediated through childhood growth. We examined data from a British cohort of 2176 women born in 1946 and for whom there were prospective measurements of birth weight and of body size throughout life. In all, 59 breast cancer cases occurred during follow-up, 21 of whom were known to be premenopausal. Women who weighed at least 4 kg at birth were five times (relative risk (RR)=5.03; 95% confidence interval=1.13, 22.5) more likely to develop premenopausal breast cancer than those who weighed less than 3 kg (*P*-value for linear trend=0.03). This corresponded to an RR of 2.31 (0.95, 5.64) per 1 kg increase in birth weight. Birth weight was also a predictor of postnatal growth, that is, women who were heavy at birth remained taller and heavier throughout their childhood and young adulthood. However, the effect of birth weight on premenopausal breast cancer risk was only reduced slightly after simultaneous adjustment for height and body mass index (BMI) at age 2 years and height and BMI velocities throughout childhood and adolescence (adjusted RR=1.94 (0.74, 5.14) per 1 kg increase in birth weight). The pathways through which birth weight is associated with premenopausal breast cancer risk seem to be largely independent of those underlying the relation of postnatal growth to risk.

It has been proposed that breast cancer may have a prenatal origin (Trichopoulos, 1990). Several studies have since used birth weight as a marker of the *in utero* environment to investigate this. Positive linear relationships between birth weight and breast cancer risk, particularly at premenopausal ages have been reported (Michels *et al*, 1996; De Stavola *et al*, 2000; Hübinette *et al*, 2001; Kaijser *et al*, 2001; Vatten *et al*, 2002; McCormack *et al*, 2003), while J-shaped associations were found in other studies (Ekbom *et al*, 1992; Sanderson *et al*, 1996; Innes *et al*, 2000; Titus-Ernstoff *et al*, 2002).

The pathways through which the foetal environment may influence breast cancer risk are not known. Birth size is a predictor of postnatal growth and adult height (Sørensen *et al*, 1999; Tuvemo *et al*, 1999; dos Santos Silva *et al*, 2002), and both age at menarche (Hsieh *et al*, 1990) and adult height (van den Brandt *et al*, 2000) are well-established risk factors for breast cancer. Thus, it is conceivable that the observed association might just be a correlate of the relation of postnatal growth with risk. Proper examination of this hypothesis has been hampered by the lack of detailed prospective data on growth throughout childhood and adolescence. Recall, by the women themselves or their mothers, of body size measurements early in life are likely to be inaccurate.

The Medical Research Council National Survey of Health and Development is a cohort of over 2000 women who have been followed since their birth in 1946 (Wadsworth *et al*, 2003). This cohort is unique in that it includes prospective measurements not only of birth weight but also of height and weight throughout childhood, adolescence and adulthood, as well as data on adult life risk factors for breast cancer. In earlier analyses of data from this cohort, we reported a positive association between birth weight and breast cancer risk and found that cases tended to be taller and slimmer throughout childhood than noncases (De Stavola *et al*, 2000). Fast height gains at ages 4–7 and 11–15 years, and steep decreases in body mass index (BMI) at ages 2–4 years, were identified as the strongest positive predictors of risk (De Stavola *et al*, 2004). In the present analysis, we have investigated whether the effect of birth weight on risk is mediated through growth in childhood and adolescence.

## MATERIAL AND METHODS

The National Survey of Health and Development is a socially stratified cohort of 5362 single legitimate live-births that occurred in Britain during the week 3–9 March 1946 and who have since been followed up. Most of the follow-up contacts were home interviews (Wadsworth *et al*, 2003), but a postal health questionnaire was also sent annually between 1993 and 2000 (from when cohort members were aged 47–54 years) to all women in the cohort with whom there was still direct contact (Kuh and Hardy, 2003). These follow-up contacts provided information on maternal age at birth, birth order, father's social class and reproductive-related variables, including menopausal status and date of menopause (defined retrospectively after 12 months of amenorrhoea), hysterectomy (or bilateral oophorectomy) and hormone replacement therapy (HRT) use. Height and weight were measured prospectively throughout childhood (at ages 2, 4, 6, 7, 11 and 14/15 years) and adulthood (at ages 36, 43 and 53 years) using standardised procedures. Leg length was derived by subtracting sitting height from standing height measured at age 43 years. Birth weight data were obtained from medical records within a few weeks of delivery. Age at menarche was reported by the girls’ mothers when the girls were 15 years old or, when this was not possible, by the respondents in the postal questionnaire at age 48 years (*n*=210). Self-reported data on breast cancer diagnosis were collected through the various follow-up contacts. In addition, in 1971, when the National Health Service Central Register (NHSCR) started to record cancers occurring in the UK population, all cohort members (including those with whom there was no longer direct contact) were ‘flagged’ at the NHSCR to provide notification of cancers, deaths and emigrations for the cohort.

Of the 2547 women in the birth cohort, 2176 (85%) who were known to be still alive on 1 January 1971 and for whom there was birth weight information and at least one height measurement in childhood, were included in the present analysis. Of the 371 who were excluded, 114 (31%) had died from nonbreast cancer causes and 176 (47%) had emigrated before 1971 and were no longer in contact with the study. A further 70 (19%) did not have any height measurements between ages 2 and 15 years and 11 (3%) had no birth weight data.

Approval for the study was obtained from all relevant ethics committees.

### Statistical methods

Breast cancer relative risks (RRs) by birth weight were estimated as rate ratios using a Cox regression model (Clayton and Hills, 1993), where age defined the time scale. Follow-up was analysed from 1 January 1971 to the earliest of date of breast cancer diagnosis, date of death or emigration, or 31 December 1999 (the last date for which the NHSCR cancer registration data were considered to be complete at the time of this analysis). The effect of birth weight on breast cancer risk was also examined separately at premenopausal ages (the women in the cohort are still too young to allow separate analysis at natural postmenopausal ages). Analyses at premenopausal ages were restricted to 1513 women who participated in recent follow-up contacts when information on menopausal status was collected. The premenopausal follow-up of these women was defined from 1 January 1971 to the earliest of: breast cancer diagnosis, date of natural menopause, date of hysterectomy (or bilateral oophorectomy), start of HRT or date of last completed questionnaire (up to 1999).

Birth weight was analysed both as a continuous and as a categorical variable (categorised into four groups: <3.000, 3.000–3.499, 3.500–3.999, ⩾4.000 kg). To assess whether the effect of birth weight on breast cancer incidence was mediated through postnatal growth, we fitted models that included birth weight plus height and BMI throughout childhood and adolescence (at ages 2–15 years) and height at age 36 years, used as a proxy for the final height achieved at the end of the adolescent growth spurt. These anthropometric variables were specified either as age-specific attained values (reflecting cumulative growth up to a given age) or as velocities between consecutive ages (capturing growth during particular age intervals). For some cohort members, childhood anthropometric data were not available at all follow-up contacts and so a multiple imputation procedure (Schafer, 1999) was used to fill in these values and obtain estimates of the birth weight effect controlled for childhood growth that were based on the whole cohort rather than the smaller subset of complete-record subjects. A full description of the multiple imputation procedure used is given elsewhere (De Stavola *et al*, 2004). Briefly, it consisted of first modelling the available measurements of height and BMI using random effects growth models that included maternal height, father's social class, birth order, birth weight, age at menarche and breast cancer incidence as explanatory variables. The model's parameters were then used to define the data distribution from which to impute the missing values. To fully account for the data variability, and assuming that data were missing at random, five sets of imputations were carried out and the results summarised as described by Schafer (1999). In models that included imputed anthropometric data, RRs were estimated as odds ratios derived from logistic regression as current multiple imputation methods cannot deal satisfactory with Cox's regression (Vach and Blettner, 2000). Owing to the completeness of the follow-up information, logistic regression produced similar odds ratios to the rate ratios obtained with Cox's regression. All RRs in Table 2 were estimated from logistic regression models. All tests of statistical significance are two-sided.

## RESULTS

In all, 59 breast cancer cases occurred during the follow-up period. The median age at incidence was 49 years (25th and 75th percentiles: 45 and 52 years). The median age at last follow-up for the remaining 2117 women was 53 years (25th and 75th percentiles: 53 and 53 years; only 3.6% were lost to follow-up, due to emigration, before reaching age 50 years). A total of 21 cases were known to be premenopausal (with ages of diagnosis ranging from 36 to 51 years) and nine were known to be natural postmenopausal at the time of diagnosis of breast cancer (age at diagnosis: 46–53 years). A further 12 had undergone hysterectomy before diagnosis or reported to have been postmenopausal at the time of diagnosis but did not indicate whether the menopause was natural. No information on menopausal status was available for the remaining 17 women. Anthropometric measures in childhood were available at each age between 76 and 90% and in adulthood for about 75% of all eligible women in the cohort. The probability that a measure was missing was unrelated to the woman's values at later ages or to her subsequent risk of breast cancer. For instance, having missing height data at age 7 years was not associated with adult height (*P*=0.24) or occurrence of breast cancer (*P*=0.67).

Table 1

**Table 1:** Breast cancer incidence rates and age-adjusted rate ratios by birth weight and age

	***N***	**No. of cases**	**Rate (per 100 000 person-years)**	**Age-adjusted rate ratio^a^**	**(95% CI)**	**Test for linear trend**
**Birth weight**						
Premenopausal ages						
*Categorical (kg)*						
<3.000	380	3	34.1	1		
3.000–3.499	559	6	46.8	1.37	(0.34, 5.47)	
3.500–3.999	469	8	74.0	2.18	(0.58, 8.21)	
⩾4.000	105	4	167.1	5.03	(1.13, 22.47)	*P*=0.03
*Per 1 kg increase in birth weight*	1513	21	—	2.37	(0.97, 5.80)	*P*=0.06
						
All ages						
*Categorical (kg)*						
<3.000	557	13	83.0	1		
3.000–3.499	802	18	80.7	0.98	(0.48, 2.00)	
3.500–3.999	652	22	120.2	1.39	(0.69, 2.77)	
⩾4.000	165	6	129.7	1.57	(0.60, 4.13)	*P*=0.21
*Per 1 kg increase in birth weight*	2176	59	—	1.46	(0.87, 2.46)	*P*=0.15

CI=confidence interval.

a As estimated from a Cox regression model (see text – Statistical methods).

shows a strong effect of birth weight on premenopausal breast cancer incidence with a steady positive trend across the four birth weight categories (*P*-value for linear trend=0.03). Women who weighed at least 4 kg at birth were five (RR=5.03; 95% confidence interval (CI)=1.13, 22.5) times more likely to develop premenopausal breast cancer than those who weighed less than 3 kg. This corresponded to a rate ratio of 2.37 (95% CI=0.97, 5.80) per 1 kg increase in birth weight. The effect of birth weight on all-ages breast cancer incidence was in the same direction, but was of a much smaller magnitude (Table 1).

**Figure 1:**
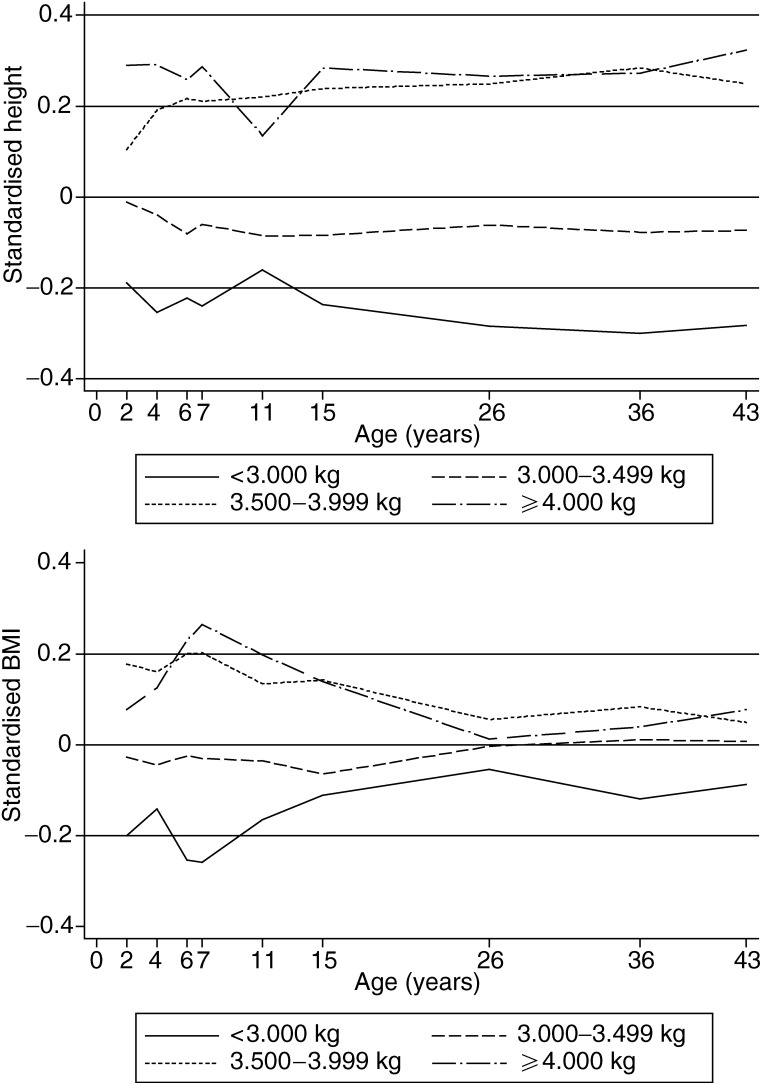
Mean standardised^†^ height and BMI by age and birth weight categories. (^†^standardised height at age *t* years=(height at age *t* years−mean (height at age *t* years))/s.d. (height at age *t* years); standardised BMI at age *t* years=(BMI at age *t* years−mean (BMI at age *t* years))/s.d. (BMI at age *t* years).

Birth weight was also a predictor of height and BMI trajectories throughout childhood and young adulthood (Figure 1

), with women who weighed 3.500 kg or more being taller and heavier throughout their childhood relative to those who were lighter at birth. Adult height (at ages 36 years), as a marker of the final height achieved at the end of the adolescent growth spurt, was associated with premenopausal breast cancer, rates increasing by 80% per every standard deviation (s.d. (about 6 cm)) increase in height (*P*=0.02). In contrast, adult BMI (at age 36 years) was inversely related to premenopausal breast cancer rates (33% reduction in rates per every s.d. (about 4 kg m^−2^) increase in BMI), but the association was not statistically significant (*P*=0.20). Similar relationships were observed with all-ages breast cancer.

The effect of birth weight on risk was only slightly reduced after adjustment for age-specific attained heights or BMIs (or interval-specific velocities) (Table 2

**Table 2:** Premenopausal breast cancer RRs per 1 kg increase in birth weight before and after adjustment for childhood height and BMI

	**Observed values**				**Observed and imputed values (*N*^a^=1513, *D*^a^=21)**	
**Controlled for**	***N*^a^**	***D*^a^**	**RR^b^**	**(95% CI)**	**RR^b^**	**(95% CI)**
None	1513	21	2.31	(0.95, 5.64)	2.31	(0.95, 5.64)
						
*Height*						
At 2 yrs	1238	19	2.20	(0.84, 5.76)	2.11	(0.85, 5.25)
At 4 yrs	1351	20	2.19	(0.84, 5.70)	2.05	(0.82, 5.11)
At 7 yrs	1347	18	2.19	(0.82, 5.86)	2.07	(0.83, 5.15)
At 11 yrs	1309	18	2.57	(0.97, 6.83)	2.16	(0.88, 5.33)
At 15 yrs	1200	16	2.01	(0.70, 5.77)	2.00	(0.80, 4.98)
Adult^c^	1345	19	1.97	(0.75, 5.15)	1.92	(0.76, 4.82)
						
Rate 2–4 yrs	1159	19	2.51	(0.96, 6.61)	2.33	(0.94, 5.76)
Rate 4–7 yrs	1227	18	2.53	(0.94, 6.80)	2.33	(0.95, 5.69)
Rate 7–11 yrs	1239	17	2.78	(1.03, 7.51)	2.32	(0.95, 5.65)
Rate 11–15 yrs	1139	16	2.27	(0.81, 6.32)	2.28	(0.94, 5.58)
Rate 15–adult^c^	1080	14	2.55	(0.86, 7.55)	2.30	(0.94, 5.61)
						
At 2 yrs+rates^d^	768	13	2.21	(0.63, 7.72)	1.87	(0.73, 4.78)
						
*BMI*						
At 2 yrs	1184	17	2.30	(0.84, 6.30)	2.44	(0.99, 5.99)
At 4 yrs	1319	20	2.46	(0.97, 6.22)	2.35	(0.96, 5.74)
At 7 yrs	1296	17	2.01	(0.74, 5.49)	2.32	(0.94, 5.71)
At 11 yrs	1292	18	2.86	(1.10, 7.45)	2.44	(1.01, 5.92)
At 15 yrs	1181	15	3.06	(1.08, 8.68)	2.43	(1.00, 5.87)
						
Rate 2–4 yrs	1092	17	2.14	(0.78, 5.90)	2.36	(0.97, 5.78)
Rate 4–7 yrs	1156	17	2.05	(0.75, 5.62)	2.30	(0.94, 5.62)
Rate 7–11 yrs	1178	16	2.31	(0.82, 6.46)	2.29	(0.94, 5.60)
Rate 11–15 yrs	1108	15	2.80	(0.98, 7.80)	2.32	(0.95, 5.64)
						
At 2 yrs+rates^e^	759	13	2.63	(0.81, 8.53)	2.38	(0.96, 5.91)
						
*Height and BMI*						
At 2 yrs+rates^f^	680	11	2.14	(0.53, 8.66)	1.94	(0.74, 5.14)

RR=relative risks; CI=confidence interval; BMI=body mass index; yrs=years.

a Number of cohort members (*N*) and breast cancer cases (*D*) for whom there was information on the relevant age-specific anthropometric variable.

b RRs as estimated by odds ratios from a logistic regression model (see text – Statistical methods).

c Height at age 36 years as a proxy for the final height achieved at the end of the adolescent growth spurt.

d Controlling for height at age 2 years and rates 2–4, 4–7, 7–11, 11–15 and 15 to adulthood.

e Controlling for BMI at age 2 years and rates 2–4, 4–7, 7–11 and 11–15 years.

f Controlling for height at age 2 years and rates 2–4, 4–7, 7–11, 11–15 and 15 to adulthood and for BMI at age 2 years and rates 2–4, 4–7, 7–11 and 11–15 years.

). Results for all women with known menopausal status (*n*=1513), using data derived from the multiple imputation procedure, showed that simultaneous adjustment for attained height at age 2 years and height velocities thereafter decreased the RR (as estimated by odds ratio from a logistic regression model) associated with 1 kg increase in birth weight from 2.31 (95% CI=0.95, 5.64) to 1.87 (95% CI=0.73, 4.78), whereas simultaneous adjustment for attained BMI at age 2 years and subsequent BMI velocities had little impact on the magnitude of the birth weight effect (RR=2.38 (95% CI=0.96, 5.91)). When both height and BMI at age 2 years and their interval-specific velocities were taken into account simultaneously, the magnitude of the birth weight effect decreased only slightly from its original value of 2.31 to 1.94 (95% CI=0.74, 5.14). Similar results were obtained using only the subsets of women for whom the relevant measurements were available, although they were based on much smaller numbers (Table 2).

Simultaneous adjustment for height and BMI at age 2 years and for height and BMI velocities throughout childhood and adolescence did not change the magnitude of the birth weight effect on all-ages breast cancer (the RR associated with 1 kg increase in birth weight before and after adjustment for growth in childhood was 1.46 (95% CI=0.87, 2.46) and 1.50 (95% CI=0.85, 2.66), respectively). Similar results were obtained when the analyses were restricted to the subset of women for whom measurements were available at all follow-up contacts.

Further adjustment for adult leg length, a better marker of growth in childhood than adult height, did not affect the magnitude of the birth weight effect on premenopausal breast cancer risk in the subset of women for whom this measure was available (*n*=720) (the RR adjusted for height and BMI velocities during childhood associated with 1 kg increase in birth weight before and after further adjustment for leg length was 2.25 (95% CI=0.62, 8.10) and 2.20 (95% CI=0.61, 7.98), respectively). Similarly, the magnitude of the birth weight effect did not change with further adjustment for age at menarche in the group of women with known age at menarche (*n*=766) (the RR adjusted for height and BMI velocities associated with 1 kg increase in birth weight before and after further adjustment for age at menarche was 2.74 (95% CI=0.75, 9.94) and 2.78 (95% CI 0.77, 10.09), respectively). Further adjustment for maternal age, birth order, father's social class, age at first birth, parity or adult BMI provided similar results.

## DISCUSSION

### Main findings

This study revealed a positive association between birth weight and risk of breast cancer, which was particularly strong at premenopausal ages. This finding is consistent with the results of an earlier analysis of data from this cohort and with those reported by other larger prospective studies (Michels *et al*, 1996; McCormack *et al*, 2003). The main contribution of the present study, however, was to show that the birth weight–breast cancer association was only reduced marginally after adjustment for measures of growth in childhood and adolescence.

### Strengths and weaknesses

This cohort is unique in that it includes prospective measurements not only of birth weight but also of height and weight throughout life. Incomplete follow-up was minimised because, as well as having frequent contacts, the cohort has been flagged through NHSCR since 1971, losses before than being essentially due to non-breast cancer deaths and emigrations at young ages. The cohort has remained representative of the native population (Wadsworth *et al*, 2003) and the number of identified breast cancer cases was similar to that expected on the basis of national incidence rates (59 observed, 57.7 expected). One of the limitations of this cohort is the small number of cases accrued so far. Information on menopausal status was available for only a subset of women in the cohort, but reanalyses censoring the information on the whole cohort at age 51 years, the median age at menopause among those with known menopausal status, provided similar results.

The value of the birth weight data as a measure of foetal growth is limited by the fact that this measure reflects both linear growth and adiposity, and by the lack of gestational age. A recent study showed stronger associations of premenopausal breast cancer with birth length than birth weight, which became even stronger after adjustment for gestational age (McCormack *et al*, 2003). Birth length for gestational age has also been shown to be a stronger predictor of adult height than birth weight for gestational age (Sørensen *et al*, 1999; Tuvemo *et al*, 1999). Although birth weight data may be subject to measurement error this is likely to have been nondifferential and, hence, it would have led to an attenuation of the true effect of birth weight on breast cancer risk.

Data on height and weight were collected prospectively throughout childhood and adulthood. A multiple imputation procedure was used to replace missing values with imputed ones, thus allowing all the analyses to be based on data from the whole cohort rather than restricting them to the various subsets for whom anthropometric data were available at each relevant age. Individual anthropometric values are likely to have been affected by random measurement errors, but the inclusion in the statistical models of all available (observed and imputed) age-specific measurements should have provided a more accurate measure of the underlying growth trajectory for each woman. No measurements were taken between birth and age 2 years, but further adjustment for the difference between a girl's standardised rank in height (or BMI) at age 2 years and her standardised rank in birth weight, this difference being taken as an indicator of early growth, did not alter the reported effects of birth weight on breast cancer risk. Although the available anthropometric data did not allow calculation of age at peak height velocity, the analysis took into account velocity of growth in early childhood and age at menarche, factors known to be closely related to age at peak height velocity (Tanner, 1989; Luo *et al*, 2003).

### Implications

It has been hypothesised that the observed associations between birth weight and breast cancer risk may reflect intrauterine exposure to oestrogens and other biological factors that may increase the number of stem cells in the breast gland and/or their proliferation (Trichopoulos, 1990, 2003). Postnatal exposure to high levels of endogenous oestrogens is known to play an important role in breast cancer in postmenopausal women (The Endogenous Hormones and Breast Cancer Collaborative Group, 2002), although the evidence is less consistent in premenopausal women (Thomas *et al*, 1997). The levels of endogenous oestrogens are about 10 times higher in pregnancy than at any other time in a woman's life (Yen, 1989) and, therefore, it is conceivable that *in utero* exposure to high concentrations of these hormones may affect the mammary tissue and the risk of malignancy later in life in the offspring (Trichopoulos, 1990, 2003). Birth weight may also be associated with breast cancer through its relationship with postnatal growth (dos Santos Silva *et al*, 2002; Sørensen *et al*, 1999). The latter interpretation would be consistent with findings from some (Hankinson *et al*, 1998; Toniolo *et al*, 2000), but not all (Kaaks *et al*, 2002), prospective studies showing that high serum levels in adulthood of insulin-like growth factor-I (IGF-I), a hormone that promotes somatic growth, are associated with an increased risk and that, similar to the effect of birth weight, the IGF-I association with breast cancer seems to be particularly stronger at premenopausal ages. Adult height, however, has been shown to be associated with both pre- and postmenopausal breast cancer (van den Brandt *et al*, 2000), suggesting that birth weight and adult height may be associated with risk through different biological mechanisms. In the present study, the magnitude of the effect of birth weight on premenopausal breast cancer risk did not change after adjustment for BMI in childhood, but it was slightly reduced after adjustment for childhood height. Thus, although the effect of birth weight on risk might, to a small extent, be mediated through childhood growth, our findings imply that birth weight affects premenopausal breast cancer risk largely through a separate biological pathway.

In short, the findings from this study suggest that the relationship between birth weight and premenopausal breast cancer risk is largely independent, rather than a correlate of the association of childhood growth and risk of this tumour.
